# Handloomed fabrics recognition with deep learning

**DOI:** 10.1038/s41598-024-58750-z

**Published:** 2024-04-04

**Authors:** Lipi B. Mahanta, Deva Raj Mahanta, Taibur Rahman, Chandan Chakraborty

**Affiliations:** 1 Mathematical and Computational Sciences Division, Institute of Advanced Study in Science & Technology (IASST) (An Autonomous R&D Institute Under Department of Science & Technology), Vigyan Path, Paschim Boragaon, P.O. Garchuk, Guwahati, Assam 781035 India; 2 Department of Computer Science and Engineering, NITTTR, Kolkata, 700106 West Bengal India

**Keywords:** Textile loom type, Handloom fabric, Powerloom fabric, Automated identification, Artificial intelligence, Deep learning, Information technology, Software

## Abstract

Every nation treasures its handloom heritage, and in India, the handloom industry safeguards cultural traditions, sustains millions of artisans, and preserves ancient weaving techniques. To protect this legacy, a critical need arises to distinguish genuine handloom products, exemplified by the renowned “*gamucha*” from India’s northeast, from counterfeit powerloom imitations. Our study’s objective is to create an AI tool for effortless detection of authentic handloom items amidst a sea of fakes. Six deep learning architectures—VGG16, VGG19, ResNet50, InceptionV3, InceptionResNetV2, and DenseNet201—were trained on annotated image repositories of handloom and powerloom towels (17,484 images in total, with 14,020 for training and 3464 for validation). A novel deep learning model was also proposed. Despite respectable training accuracies, the pre-trained models exhibited lower performance on the validation dataset compared to our novel model. The proposed model outperformed pre-trained models, demonstrating superior validation accuracy, lower validation loss, computational efficiency, and adaptability to the specific classification problem. Notably, the existing models showed challenges in generalizing to unseen data and raised concerns about practical deployment due to computational expenses. This study pioneers a computer-assisted approach for automated differentiation between authentic handwoven “*gamucha*”s and counterfeit powerloom imitations—a groundbreaking recognition method. The methodology presented not only holds scalability potential and opportunities for accuracy improvement but also suggests broader applications across diverse fabric products.

## Introduction

### Context

**Figure 1:**
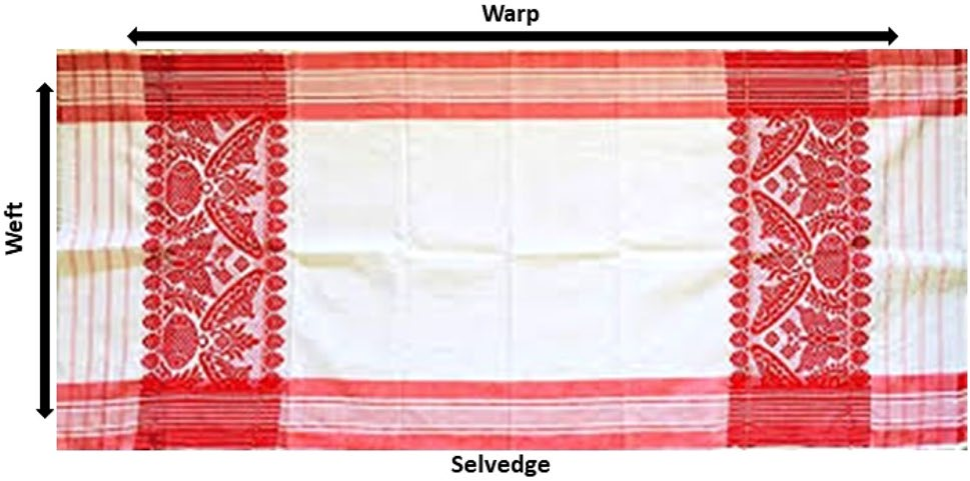
The “*gamucha*”.

The pride and protection of handloom heritage are sentiments shared by many countries across the world. Handloom traditions represent an essential part of a nation’s cultural identity and history, and they are revered for their artistic craftsmanship and time-honored techniques. In the textile industry, India plays an important role where it contributes to 15%^1^ of the total Industrial production and nearly 30% of the total exports^2^. In fact, it is the second largest employment generator after agriculture^3^. The textile sector in India encompasses modern textile mills, independent powerlooms, handlooms, and garments. Handloom holds significant economic importance, particularly for traditional products like the renowned “*gamucha*” towel from Assam, India (Fig. 1), valued not only for its utility but also cultural symbolism. It is a white rectangular piece of cotton hand woven cloth with primarily a red (in addition to red, other colors are also used) border on two/three sides (longer side) and red woven motifs on the one/two sides (shorter sides).

**Figure 2:**
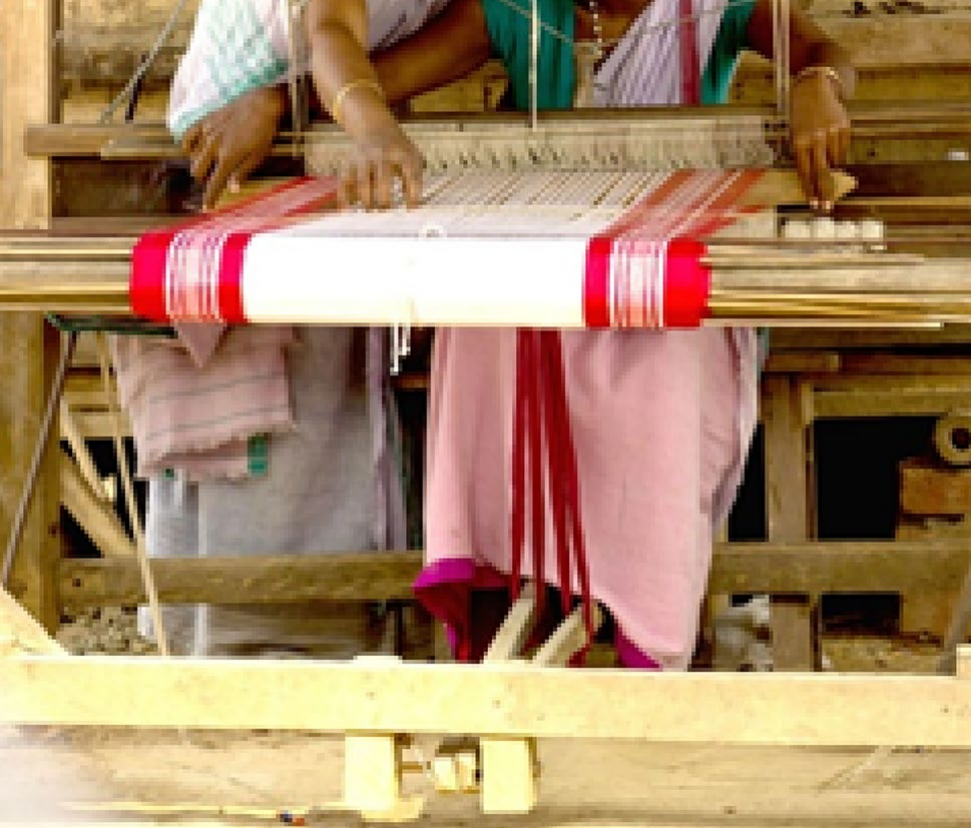
Women weavers in Assam weaving in a traditional wooden-made loom.

Despite its cultural significance, the handloom industry faces a severe crisis, exacerbated by competition from powerlooms selling products deceptively as handloom items^3–5^. The 2019–2020 Indian handloom census revealed Assam to have the highest number of weavers’ families, with 10.9 lakh (38.6%) households, predominantly in rural areas^6,7^. Unfortunately, the crisis has disproportionately affected female weavers, constituting nearly 80% of the handloom workforce in the state^1^ (Fig. 2). Weavers, integral to Assam’s villages, face financial and cultural challenges.

Efforts by the State’s Handloom and Textile Directorate, including raids on powerloom products, have been hampered by the lack of valid laboratory certificates to support complaints, leading to the release of confiscated items back to retailers or wholesalers^6,7^. Addressing this issue is crucial to preserving the handloom heritage and supporting the livelihoods of the weaver community^4–6^.

### Mechanism of handloom “*gamucha*” fabric production

Fabric production begins with yarn, crafted from various fibres, either natural or man-made. Although the “*gamucha*” is woven in various yarns, like silk, wool, etc., the cotton thread or yarn is used most commonly. After passing through stages, yarn is derived from these fibres, which must possess specific properties to qualify as textile fibres. Fabric, typically woven, requires two thread series: warp (longitudinal) and weft (transverse). Weaving involves interlacing these series in a loom. However, before weaving, the longitudinal warp threads undergo preparatory processes. Yarn is often in hank form, unsuitable for warping. Preparatory weaving processes include sizing, winding, warping, beaming, looming, and finishing. Sizing involves coating the yarn for strength and uniformity. Winding transfers yarn from hank to bobbin. Warping involves preparing the warp sheet, followed by denting to draw threads through reed dents. Beaming transfers the warp sheet to the warp beam. The final steps include heald (a device used in weaving looms to control the movement of warp yarns) knitting and looming, where the shuttle passes through the warp-shed, completing the weaving process. This comprehensive process ensures the creation of high-quality handloom “*gamucha*” fabric production.

### Powerloom “*gamucha*” and challenges thereof

The production of high-quality handloom “*gamucha*” demands significant skill and time from weavers, resulting in a meticulous process. In contrast, powerloom counterparts can be mass-produced at a lower cost due to the use of cheaper yarns. The challenge arises due to the subtle differences between the two types, making it difficult for both non-experts and even experts to distinguish them without scientific support.

Experts rely on manual observations of features. Few common differences observed are:

(i) Fabric Feel: Handloom “*gamucha*” feels softer due to the use of pure, handmade yarns, while powerloom versions tend to be stiffer as they often utilize synthetic yarns like polyester for cost efficiency and durability.
(ii) Weft Uniformity: Handloom “*gamucha*” displays uneven pick-ups, varying from weaver to weaver, whereas powerloom versions exhibit consistent pick-ups.
(iii) Occasional lumps: Handloom “*gamucha*” fabric has occasional lumps due warp breakage and repairing.
(iv) Selvedge Markings: Handloom “*gamucha*” selvedges bear distinct temple marks, absent in powerloom counterparts.
(v) Thread Type: Handloom “*gamucha*” predominantly employs twisted threads or yarns, while powerloom versions utilize single untwisted yarns.

**Figure 3:**
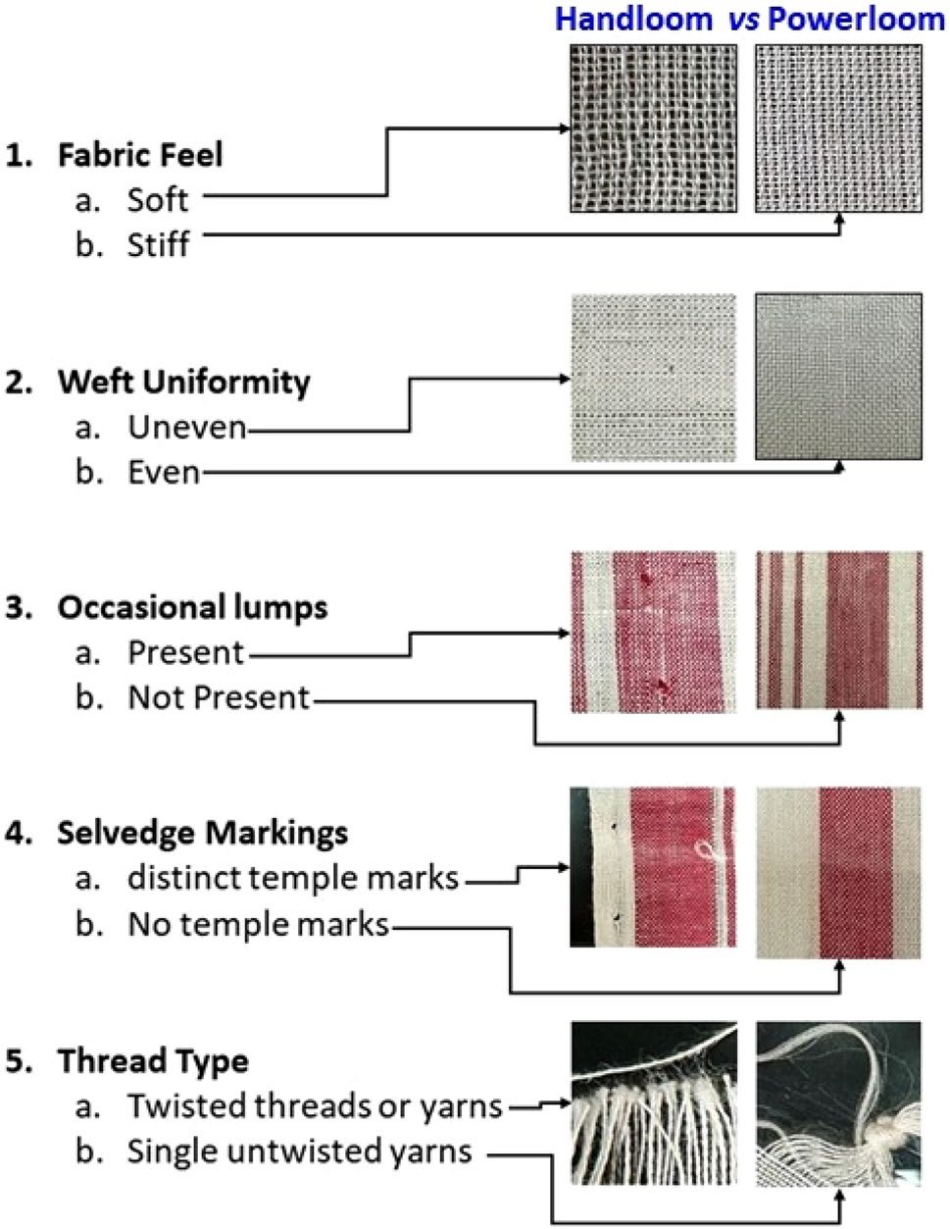
Some of the important handcrafted features for identification “*gamucha*” handloom.

**Figure 4:**
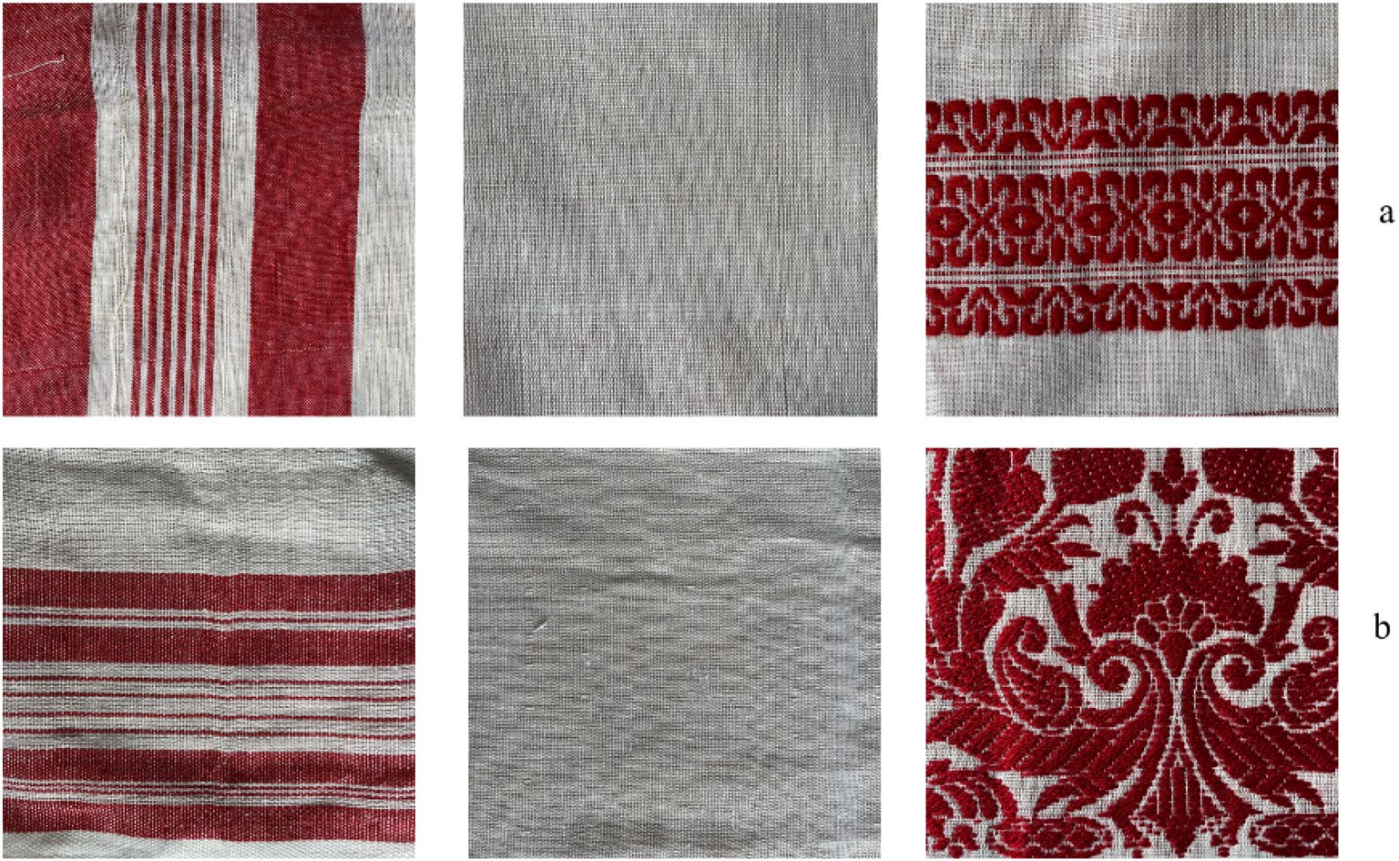
Images acquired of the two loom type of the “*gamucha*” (**a** handloom; **b** powerloom).

These observations span different sections of the “*gamucha,*” including selvedge and short edges, inner body, and motifs. Images from all these sections contribute to the identification of the loom type. Figure 3 visually outlines features crucial for expert manual identification. All these images have been captured using the iPhone 12 smartphone in 4x zoom and in natural light. Further, in Figure 4, images of handloom “*gamucha*” (row a) and powerloom “*gamucha*” (row b) are presented for comparison. Placing similar sections of the cloth one above the other underscores the challenge of distinguishing between them, emphasizing the need for a systematic approach, such as the proposed automated recognition system, to address this complexity.

### Artificial intelligence (AI) in the textile industry

Within the landscape of the Fourth Industrial Revolution (IR4.0), AI emerges as a cornerstone in the textile industry, significantly enhancing the quality of textiles^8–11^. Its pivotal role lies in its capacity to adeptly identify defects, thereby contributing to the overall improvement of textile standards. It is gaining prominence, particularly in the areas of loom type detection and fraud prevention. AI-driven technologies, such as computer vision, play a pivotal role in accurately identifying various loom types, streamlining manufacturing processes, and ensuring quality control. Additionally, AI’s advanced analytics capabilities are instrumental in detecting fraudulent claims within the industry, mitigating risks and ensuring transparency. By harnessing AI for loom identification and fraud prevention, the textile sector not only enhances operational efficiency but also establishes a foundation for trust and integrity within the supply chain.

### Rationale of the study

The significance of this study lies in its potential to assist handloom experts in their identification process, addressing a critical need in the industry. By incorporating AI technologies, specifically deep learning models and transfer learning architectures, we aim to classify handloom “*gamucha*”s from power loom counterparts with cotton yarn type. Our study introduces a novel deep learning model for automated loom type identification, filling a gap in existing literature and representing a pioneering effort in this domain. By addressing these aspects, the research endeavors to contribute not only to the technological advancement of the textile industry but also to the preservation and sustainability of traditional handloom practices in the face of contemporary challenges.

The contribution of this study can be bulleted as follows:

- Creation of a meticulously benchmarked image dataset featuring “*gamucha*” fabric from handloom and powerloom sources.
- Pioneering the development of an innovative deep learning model specifically designed for the precise identification of handloom “*gamucha*”s amidst powerloom counterparts.
- Crafting a seamlessly user-friendly and immediately deployable application tailored for the seamless implementation of the developed model, ready for translation or further study across diverse contexts.

## Prior art

The application of AI in the domain of textile fabrics has alluded attention, although being a crucial one. It is observed that the first phase of works was initiated in 2005^12^, where porosity calculation was done on 30 microscopic images of plain woven cotton fabrics. To assess the textile porosity by the application of the image analysis techniques, it was revealed by the authors that light transparency of the looser fabrics is higher than that of the tighter ones because of the more significant pore dimensions. The subsequent study was reported in 2010^13^, where the authors employed Discrete wavelet transform, and the first-order statistical features, such as mean and standard deviation, are obtained and stored in a library. The obtained value is compared with the reference image value for determining any kind of defects on the fabric. Here, the study aimed to identify defects in a handloom silk fabric using image analysis techniques.

In 2011, a study on fabric texture analysis was done using the computer vision technique^14^. The other study^15^ presented an application of machine learning to distinguish between different materials such as carpet, flooring vinyl, tiles, sponge, wood, and polyvinyl-chloride (P.V.C.) woven mesh based on their surface texture. Several machine learning algorithms, such as Naive Bayes, decision trees, and naive Bayes trees, have been trained to distinguish textures sensed by a biologically inspired artificial finger.

The subsequent development^16^ was reported in 2014, where the authors developed a novel structure detection method based on Radon transform using high-resolution images of fabric yarn patterns. Applied on three kinds of yarn-dyed cotton of 24 samples of microscopic images, it was shown that the edge-based projection method performs better than the gray projection method, especially when there is long hairiness on the fabric surface for identification of warp and weft. Using texture feature for textile image classification was further provided in 2015^17^, using 450 different textured images of different cloth material with variant design. The authors have used feature extraction methods G.L.C.M., Local binary pattern, and moment invariant (MI). Then feature reduction is performed using P.C.A., followed by classification using SVM. The accuracy achieved is 74.15%.

In 2016, Jing et al.^18^ worked on fabric defect detection on the T.I.L.D.A. database using Gabor filters for feature extraction, followed by feature reduction kernel P.C.A. Euclidean normal and OTSU is used for similarity matrix calculation. The sensitivity, specificity, and detection success rate are measured and reported to be 90% to 96%. Specificity is in the range above 96%, and the detection success rate is above 93% for different defect types. 2017 saw another novel biologically-inspired method^19^ to invariantly recognize the fabric weave pattern (fabric texture) and yarn color from the color image input. The authors proposed a model in which the fabric weave pattern descriptor is based on the H.M.A.X. model for computer vision inspired by the hierarchy in the visual cortex. The color descriptor is based on the opponent color channel inspired by the classical opponent color theory of human vision. The classification stage is composed of a multi-layer (deep) extreme learning machine.

In 2018, Huang et al.^20^ performed a study on textile grading of fleece based on pilling assessment using image processing and machine learning methods. Three hundred twenty representative samples were collected from fabrics and classified as grade 2, 3, 4, or 5—each grade comprised 80 samples. The obtained grayscale images were filtered using two methods: the DFT method combined with Gaussian filtering was used to smooth the grayscale images. ANN and SVM are used for classification. Classification accuracies of the ANN and SVM were 96.6% and 95.3%, respectively, and the overall accuracies of the Daubechies wavelet were 96.3% and 90.9%, respectively. Again, another work^21^ provided a literature review on the application of data mining and machine learning in the textile industry.

In a 2023 study by Bora et al.^22^, a methodology employing a Machine Learning (ML) classifier and utilizing a database of 7200 images from handloom and powerloom types achieved a notable 97.83% accuracy in automated loom recognition. The approach involved extracting texture features, employing significant ones based on a t-test, and training using all possible feature combinations. Precision rates were 97% (handloom) and 98% (powerloom), with recall rates of 98% (handloom) and 97% (powerloom). Notably, the study focused only on digital camera images and lacked validation results.

## Methodology

### Materials used

**Figure 5:**
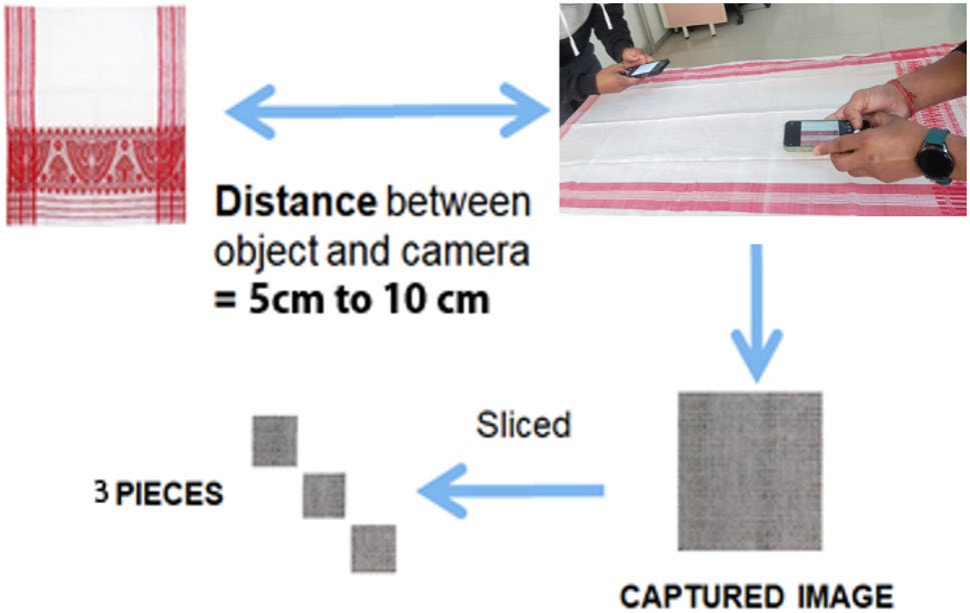
Image acquisition method.

For our study, we obtained high-resolution images of segments from “*gamucha*”s using a predetermined methodology (as depicted in Fig. 5). Specifically, we captured images from 200 pieces, with an equal distribution of 100 from handloom and 100 from powerloom classes. Two different smartphone models (iPhone 12 and Xiaomi 11i) were used to address source variation. External factors such as illumination (pictures were taken without flash), focus (we manually observed focus by tapping on the phone), and distortion (we tapped on the phone along a bent line, if present, and held until the line straightened automatically) were taken into account during image capture, maintaining a distance of 5–10 cm from the fabric. Detailed camera specifications are provided in Table 1.

**Table 1:** Specific details about the camera used.

Model	Zoom factor	Pixels	Resolution
iPhone 12	1x	3000 × 3000	9,000,000 pixels or 9 megapixels
Xiaomi 11i	1x	3024 × 3024	9,144,576 pixels or approximately 9.14 megapixels

**Figure 6:**
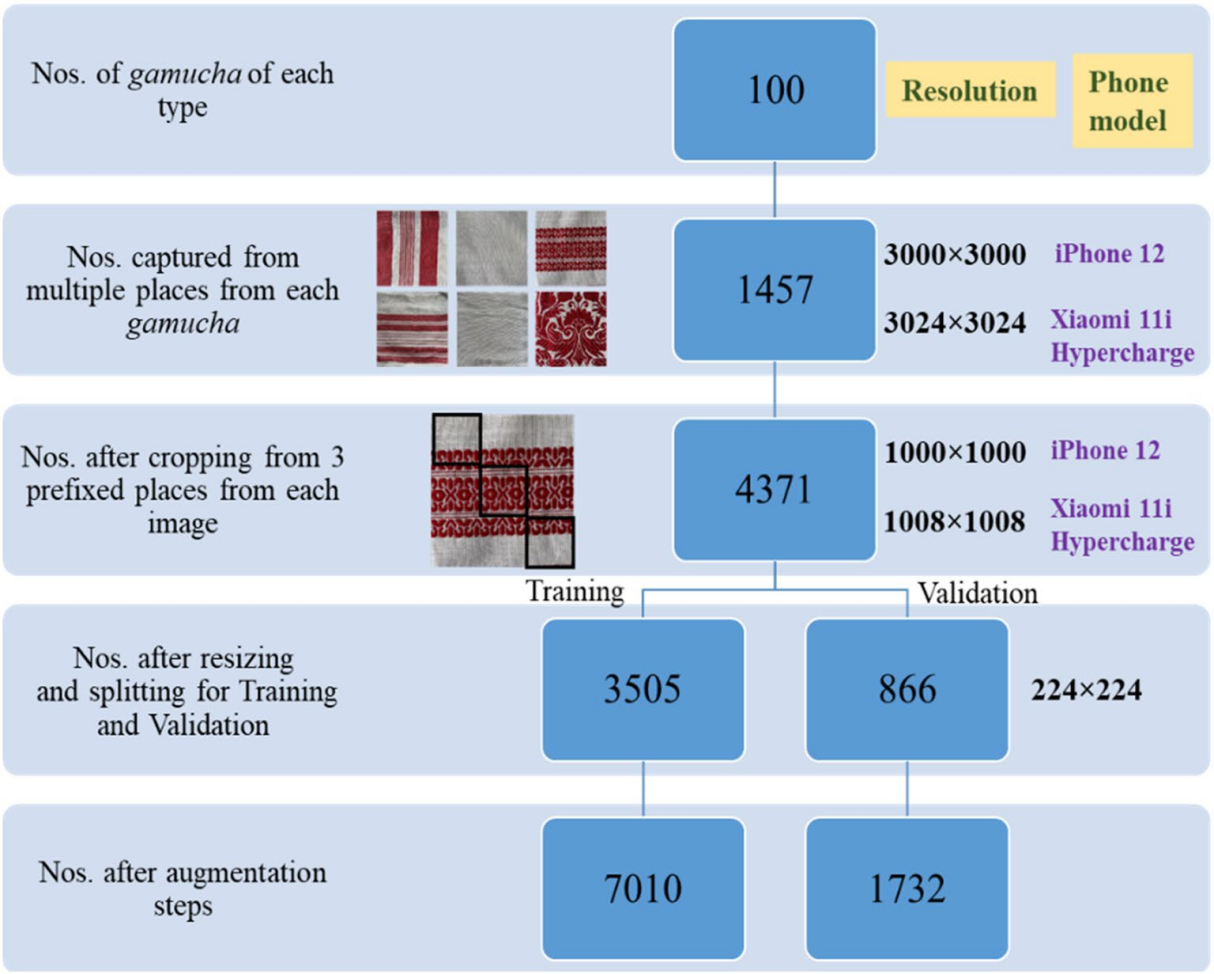
Dataset preparation flow.

By following a systematic dataset curation flow, bulleted below and depicted in Fig. 6, we ensured the representation of various features of “*gamucha*”s in our dataset, preparing it for training and validation in the development of a smartphone-based app.

- Source Validation: “*Gamucha*”s were collected from various weaving centers for handloom and seized stock for powerloom, validated by experts from the Department of Handloom and Textile, Govt. of Assam, ensuring reliable groundtruth.
- Image Capture: Multiple images were captured from each “*gamucha*” to represent various features, totaling 1457 images for each type.
- Cropping and Resizing: Three equal square sections (top-left, bottom-right corners, and center) were cropped from each image to offer diverse perspectives. After resizing to 224 × 224 (to match model specifications) the dataset comprised 4371 images per class.
- Data Split: Of these, 3505 images per class were allocated for training, and 866 were set aside for validation.
- Image Augmentation: Image augmentation techniques were applied to enhance dataset diversity and model generalization, as follows:
  i. *Rescaling*: rescale = 1.0/255 is used to normalize the image pixels. This rescales the RGB pixel values from [0, 255] to [0, 1].
  ii. *Rotation*: rotation_range = 180 randomly rotates the image within the range of 0 to 180 degrees. This simulates the effect of the object being oriented differently.
  iii. *Vertical Flip*: vertical_flip = True randomly flips the image vertically (upside down). This assumes that the object classification is not affected by vertical orientation.
  iv. *Horizontal Flip*: horizontal_flip = True randomly flips the image horizontally (mirror image). This is useful when the object classification is invariant to horizontal flipping.
  v. *Brightness Range*: brightness_range = [0.5, 1.5] randomly changes the brightness of the image by a factor between 0.5 to 1.5. This simulates varying lighting conditions.
  vi. *Zoom Range*: zoom_range = [1, 1.5] randomly zooms into the images up to 50%. This could help the model learn to recognize objects even when they occupy different amounts of space within the image.

No picture enhancer software was employed for additional processing of the captured images, ensuring a true-to-life acquisition to the greatest extent possible.

Following augmentation, the number of training images doubled to 7010, and the validation images increased to 1732 for each class. Hence, the final dataset consisted of 17,484 (14,020 training and 3464 validation) images.

### Problem definition

Defining this problem, we may proceed as follows:

Let $G={\left\{{g}_{k}, {s}_{k}\right\}}_{k=1}^{N}$ be a set of “*gamucha*” images with corresponding scores, $I$ be the image space, and $S$ represents the possible scores set. Here, $N$ is the number of samples, ${g}_{k}\in I$ and ${s}_{k}\in S$. Our work aims at performing binary classification and thus, provides a prediction score ${s}_{k}$ for the given image ${g}_{k}$. To train our model $i$, a training set $T\subset {\text{G}}$ is chosen and is used to make the model learn the following mapping:

1 $${\varnothing }_{D{L}_{ar}}^{i}:G\to S$$

where $i\in \left[\mathrm{1,6}\right]$ denotes the six deep learning models and ${\varnothing }_{D{L}_{ar}}$ represents the mapping function related to the selected models that are composed of a feature extractor and a linear layer with binary output logits. Besides focusing on providing an efficient solution to the stated classification problem, this paper conducts a comparative analysis on ${\varnothing }_{D{L}_{ar}}$ of various deep learning architectures to bring forth conclusive results.

### Methods (deep learning models)

With the emergence of deep learning techniques, textile engineering has adopted deep networks for providing solutions to classification-related problems. These include classification based on fabric weaving patterns, yarn colors, fabric defects, etc.^19,23^. We investigated the performance of six deep learning architectures, which include VGG16^24^, VGG19^24^, ResNet50^25^, InceptionV3^26^, InceptionResNetV2^27^, and DenseNet201^28^. Each model is trained with annotated image repositories of handloom and powerloom “*gamuchas*”. Consequently, the features inherent to the fabric structures are ‘learned’, which helps to distinguish between unseen handloom and powerloom “*gamucha*” images. Deep learning obviates the requirement for independent feature extraction by autonomously learning and discerning relevant features directly from raw data. This inherent capability streamlines the process, enhancing adaptability to diverse datasets and eliminating the need for manual feature engineering.

**Figure 7:**
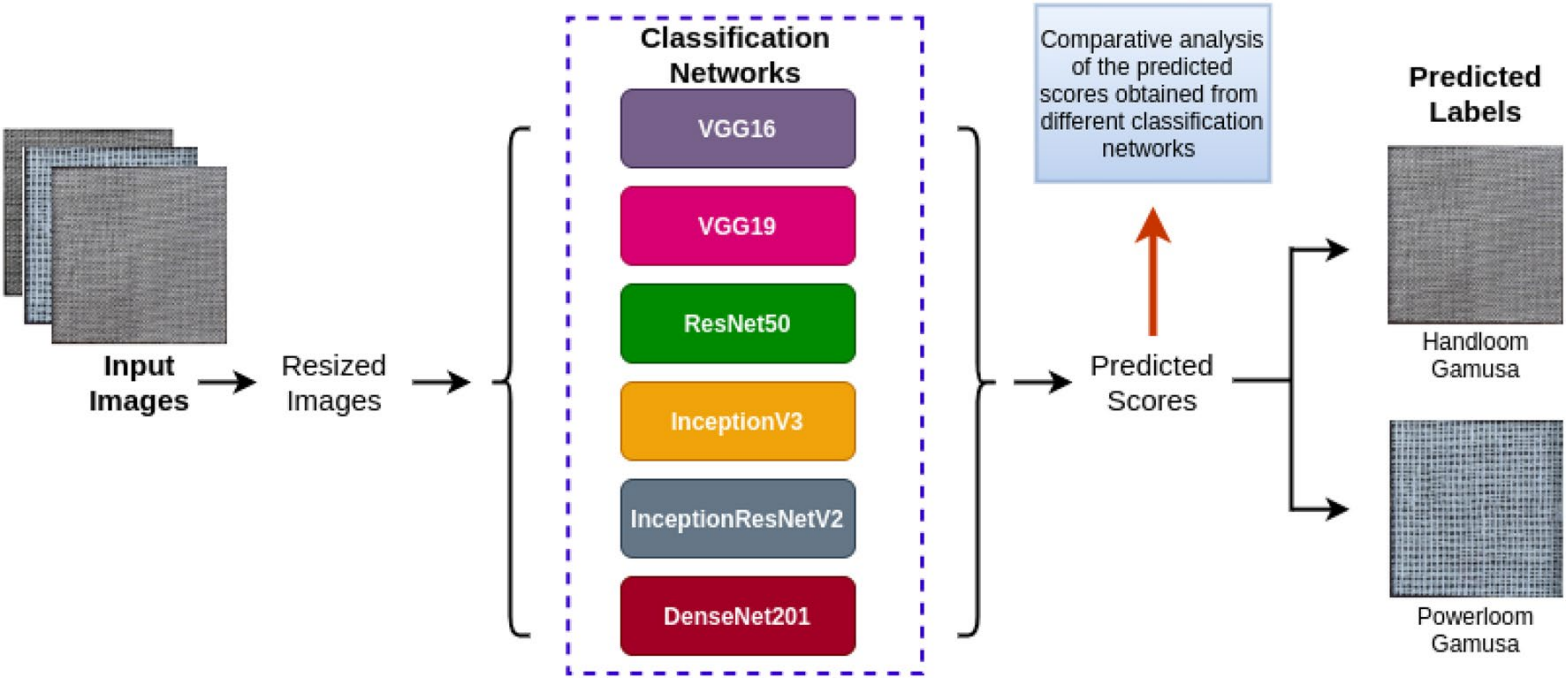
Working principle of the proposed models.

As shown in Fig. 7, six deep learning models are employed to perform classification with the objective of differentiating handloom “*gamucha*”s from powerloom “*gamucha*”s. The technical and architectural description of each model is provided below:

#### VGG neural networks

V.G.G. is a convolutional neural network (CNN) architecture which addresses the important aspect of depth in deep networks and uses small convolutional filters, allowing the model to have a large number of weight layers. Our work considers the two most successful deep CNNs proposed by VGG-VD, namely VGG16 and VGG19 with 16 and 19 weight layers, respectively. Both these networks use a stack of 3 × 3 kernel-sized filters with stride 1, thus presenting a small receptive field. These are further followed by multiple non-linearity layers. This contributes to increasing the network’s depth and helps learn more complex features with discriminative decision functions. This architecture proved to be a tremendous breakthrough in image classification with an achievement of 92.7% top-5 test accuracy in the ImageNet dataset^29^. Liu et al.^30^ experimented with VGG16 and its variants and concluded their effectiveness in detecting complicated texture fabrics. Considering this analysis in the textile domain, we adopted VGG16 and VGG19 for our classification problem.

#### Residual neural network (ResNet)

In^25^, the concept of residual networks was introduced, emphasizing the vanishing gradient problem in deep networks that causes learning to be negligible at the initial layers in the backpropagation step. The deep ResNet configuration overcomes this issue by employing a deep residual learning module via additive identity transformations. ResNet is the winner of the classification task in the ILSVRC-2015 competition and has been used as a basic structure in many fabric recognition and classification applications^23,31^. Inspired by the performance of ResNet in these domains, we experimented with ResNet50. It is a variant of the ResNet model, which has 48 convolutional layers along with 1 max-pooling and 1 average-pooling layer.

#### Inception

Inception networks were introduced by GoogleNet, which are proved to be more computationally efficient, both in terms of the number of parameters generated by the network and the economic cost incurred (memory and other resources). Inception v3 is the third version of the series with additional factorization convolutions, aiming to reduce the number of parameters while maintaining network efficiency. In addition to this, several other techniques for optimizing the network have been suggested to loosen the constraints for more straightforward model adaptation. These techniques include regularization, dimension reduction, and parallelized computations. The model comprises different sized filters at the same layer, which helps obtain more exhaustive information related to variable-sized patterns. Moreover, Inception v3 is widely adopted in image classification tasks^32,33^ and is proved to achieve 78.1% accuracy with ImageNet Dataset and top-5 accuracy about 93.9%.

#### Inception with residual blocks

InceptionResNetV2 is the hybrid network that integrates the two high-performing CNNs, namely ResNet and Inception. Its configuration comprises residual connections that add up the output of the inception modules to the input. This allows to increase the number of inceptions blocks; accordingly, the network depth also increases. The batch normalization is used only on top of the traditional layers rather than on the summation. When considering the computational cost, it is estimated to be similar to that of Inception v4.

#### DenseNet

DenseNet architecture is designed in such a way that it contributes towards solving vanishing gradient problems due to network depth. Specifically, all layers’ connection architecture is employed, i.e., each layer acquires inputs from all previous layers and conveys its own feature maps to all subsequent layers. This ensures the maximum flow of information between layers. This network architecture removes the necessity to learn redundant information, and accordingly, the number of parameters is significantly reduced (i.e., parameter efficiency). It is also efficient for preserving information owing to its layers’ connection property. DenseNet201, a specific implementation under this category with 201 layers’ depth, is used in this paper to study its potential in classifying “*gamucha*” images.

#### Proposed model

In our pursuit to differentiate between handloom “*gamucha*” and powerloom “*gamucha*”, we devised a custom model named “gamucha.AI”.

Our model for the classification of the images was built on the VGG 16 transfer learning architecture, explained earlier. This model was selected for the base model because we wanted lesser layers in the architecture, a characteristic of VGG 16. First the model was not modified. Next, in order to improve the model some modifications were made. In the first modification the last three dense layers of the original VGG16 architecture were dropped and replaced with a few slightly modified dense layers. Using transfer learning, these newly added layers were trained while keeping the weights of the remaining layers frozen. However, it displayed overfitting. In the second modification, to avoid overfitting, the final dense layer of the model was retrained with data augmentation with a dropout layer added between the last two dense layers. This modification addressed overfitting but displayed bias.

**Figure 8:**
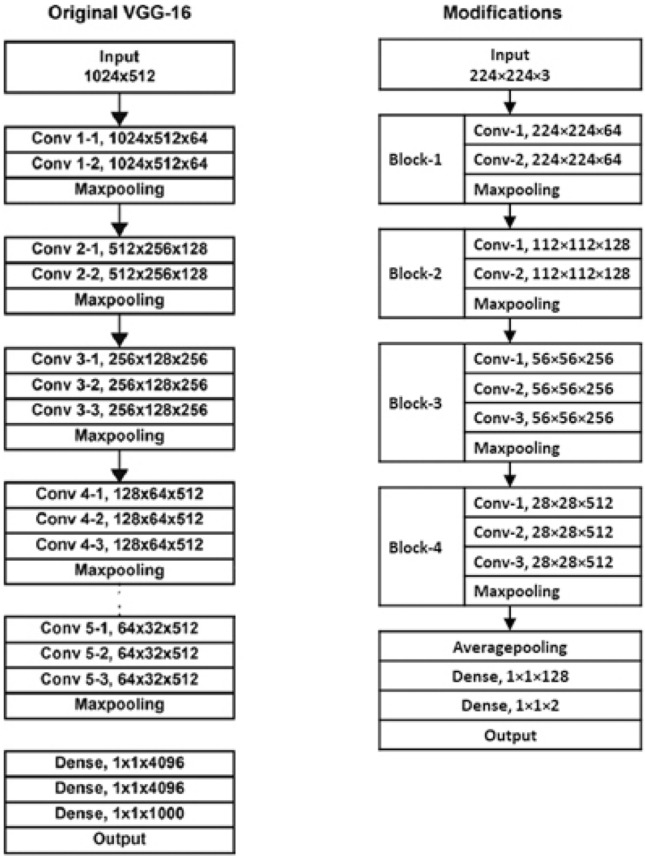
The architecture of the proposed model “gamuch.AI”.

To avoid the issue, a third modification was done and it was decided to train the entire model, instead of using transfer learning. The input images for this model were standardized to a size of 224 × 224, specifically cropped images. This choice was deliberate, as larger sizes were escalating model complexity, while smaller dimensions, i.e. below 224 × 224, resulted in information loss. Thus, 224 × 224 emerged as the optimal size for achieving a balance between model simplicity and information retention. However, since the amount of training data available was limited, it was decided to reduce the complexity of the original VGG16 architecture. Hence, the fifth convolutional block of the original VGG16 architecture was removed and an average pooling layer was added, followed by the two dense layers. To avoid overfitting, data augmentation was used with several augmentation techniques such as rotating, vertical flipping, zooming and different brightness levels. Zooming in on the images allows the model to noticeably identify the loop structure of the images. The final proposed model is depicted in Fig. 8.

The entire model was trained from scratch, using the Adam optimizer, at a learning rate of 0.00001.

### Loss definition

As stated before, we aim to analyze different ${\mathrm{\varnothing }}_{D{L}_{ar}}^{i}$ belonging to the architectures mentioned above that automatically predict the score ${s}_{k}\in S$. According to the problem definition specified in Section "Problem Definition", ${s}_{k}$ determines whether the outcome belongs to the positive class (handloom “*gamucha*”s) or the negative class (powerloom “*gamucha*”s). The predicted probabilities of the two classes can be defined by ${p}_{i}\in \left[\mathrm{0,1}\right]$. Considering the binary classification task, we opted for the binary cross entropy (B.C.E.) loss function, which can be given as:

2 $$BCE=-\frac{1}{TN}\sum_{i=1}^{TN}{y}_{i}log\left({p}_{i}\right)+{(1-y}_{i})log\left({1-p}_{i}\right)$$

where $TN$ is the training image sample space.

### Evaluation metrics

Apart from Accuracies and Losses, we used the following metrics to measure our classification task: Precision, Recall, Sensitivity, Specificity, and F1-score. The precision determines the effectiveness of the model incorrectly recognizing the actual labels. Sensitivity takes into account the potential of the system in detecting the positive class. Both precision and sensitivity are fused into one numerical value, called F1-score. Specificity focuses on the proportion of negative samples correctly classified. As mentioned earlier, each of the evaluation metrics computed for all the models is individually studied to provide a robust solution for our specified classification problem.

### Platform used for model development

Experiments and implementations are done with using python with the TensorFlow framework and Keras API.

### Platform used for mobile application

An initial prototype of the mobile application is developed using Flutter. Flutter is an open-source UI software development kit created by Google. It is used to develop cross-platform applications for Android, iOS, Linux, macOS, Windows, Google Fuchsia, and the web from a single codebase.

The workflow of the proposed Mobile Application is as follows:

1. Image Capture: The app captures 3 pictures of the “*gamucha*” using the phone’s camera, ensuring a distance of 5–10 cm for optimal image quality.
2. Automatic Upload: After clicking on “Analyse now”, the captured images are automatically uploaded to the processing server through an API, facilitating seamless data transfer.
3. Server-side Preprocessing: The uploaded images undergoes preprocessing (cropped and resized) on the server to enhance its quality and prepare it for input into the AI model.
4. AI Model Inference: Each preprocessed image is fed into a trained AI model hosted on the server for prediction, leveraging the model’s learned patterns and features.
5. Individual Accuracies: Accuracies for each of the three cropped images are calculated separately, providing insights into the model’s performance on individual instances.
6. Average Result Calculation: The final prediction is determined by calculating the highest-predicted result from the predictions of the three images, promoting a more robust and balanced outcome.
7. Result Display to User: The final prediction is sent back to the mobile app and displayed to the user, ensuring transparency and user engagement.
8. No Image Storage: Emphasizing user privacy, no images are stored on the server, aligning with data protection principles.
9. Automatic Image Removal: Once the prediction process is complete, all uploaded images are automatically removed from the server, maintaining data hygiene and minimizing storage concerns.

## Results

### Implementation and comparative analysis of different deep learning architectures

This subsection presents experimental results and comparative analysis to conclude with the best model among the selected classification networks—this aids in obtaining an efficient solution for our stated problem.

**Figure 9:**
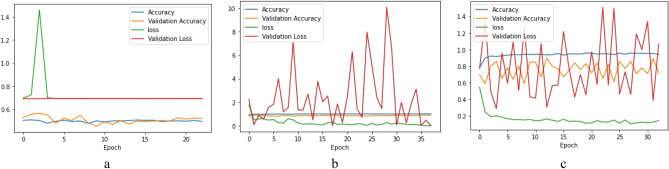
(**a**) VGG16 Model training curve. (**b**) VGG16 Model training curve with transfer learning. (**c**) VGG16 with transfer learning and data augmentation.

Implementing the VGG16 architecture the accuracy arrived was around 50% to 56%. The training curve is shown in Fig. 9a. Implementing the first modification, the model reached a maximum training accuracy of 99.94% and a validation accuracy of 91.99%, as revealed in the training curve in Fig. 9b. Next, implementing the second modification, the model reached a training accuracy of 95% and a validation accuracy of 90% after 15 epochs. and didn’t seem to be further improving, as the training curve (Fig. 9c) depicts.

**Figure 10:**
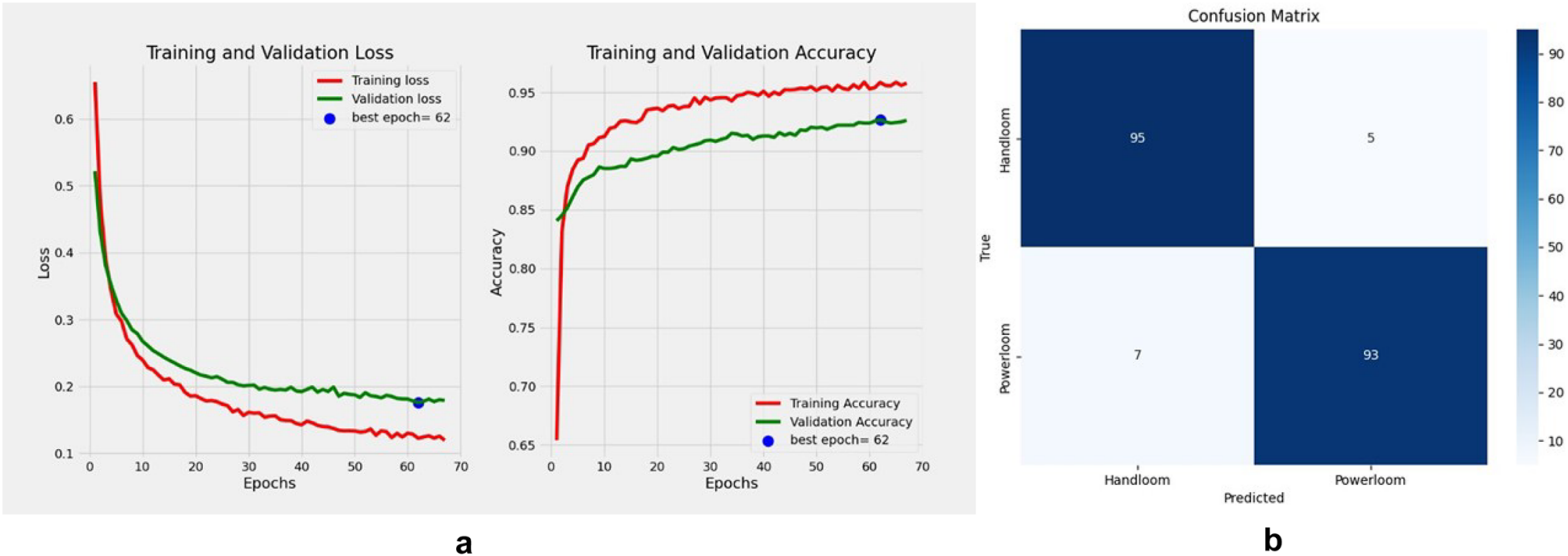
(**a**) VGG16 Model training curve after removing the 5th block and adding new layers (**b**) confusion matrix.

Finally, implementing the third modification, the model achieved a training accuracy of 98.47%, and a validation accuracy of 94.39%, after 43 epochs. This model was then tested on 25 unknown images of each type each, which were augmented (horizontal flip, vertical flip and mirroring the horizontal flip, vertical flip) to 100 images each type. The accuracy obtained was 98.0%, revealed in the training curve Fig. 10a. The confusion matrix is depicted in Fig. 10b.

It was seen that some powerllom “*gamucha*” were wrongly identified as handloom. These images were identified and checked and were found to be blurry, indicating that the images have to be well focused before running the model.

### Comprehensive overview of model characteristics

In the context of deep learning models, “space occupancy,” “number of parameters,” and “associated depth” refer to different aspects of a model’s architecture and characteristics:

*Space Occupancy*: Space occupancy typically refers to the memory or storage requirements of a deep learning model. It represents the amount of memory needed to store the model’s parameters and other information required for inference or training. This is usually measured in terms of megabytes (MB) or gigabytes (GB) of memory. A model with high space occupancy requires more memory for its operation.

*Number of Parameters*: The number of parameters in a deep learning model refers to the total count of learnable weights and biases that the model uses to make predictions or classifications. In neural networks, these parameters are typically associated with the connections between neurons in different layers. More parameters often allow a model to capture more complex patterns in data, but they also increase the computational and memory requirements.

*Associated Depth*: The depth of a deep learning model refers to the number of layers it has. Deeper models have more layers, and each layer typically performs a specific transformation of the input data. Deeper models can capture more intricate hierarchical features in the data, but they may also be more challenging to train and require more computational resources.

These three aspects are interconnected in deep learning models. As we increase the depth and the number of parameters, we often increase the space occupancy, as more memory is required to store the additional parameters. In machine learning and neural networks, non-linearity refers to the capability of a model to capture complex relationships between input and output variables beyond simple linear functions. In the context of classifying *‘gamucha*’ images into handloom and powerloom categories, ResNet50, VGG16, and VGG19 offer a good balance between performance and computational cost due to their moderate depth, as observed in Table 2. Deeper models like InceptionV3, InceptionResNetV2, or DenseNet201 can provide even higher accuracy due to their increased depth and non-linearity. However, it’s essential to strike a balance, as excessively large models may lead to overfitting on the training data and require substantial computational resources for training and inference.

**Table 2:** Model characteristics comparison.

Model	Model size (space occupancy) (MB)	Number of parameters	Model depth (associated depth)
ResNet50	1722.54	451,423,106	179
VGG16	512.26	134,268,738	23
VGG19	532.53	139,578,434	26
InceptionV3	948.06	248,311,586	315
InceptionResNetV2	873.34	228,416,738	784
DenseNet201	1605.81	420,467,266	711
Proposed Model	41.44	10,846,914	19

Our lightweight model demonstrates remarkable performance while maintaining computational efficiency, marking a significant achievement, especially considering its intended integration into a smartphone application.

### Comparison

**Figure 11:**
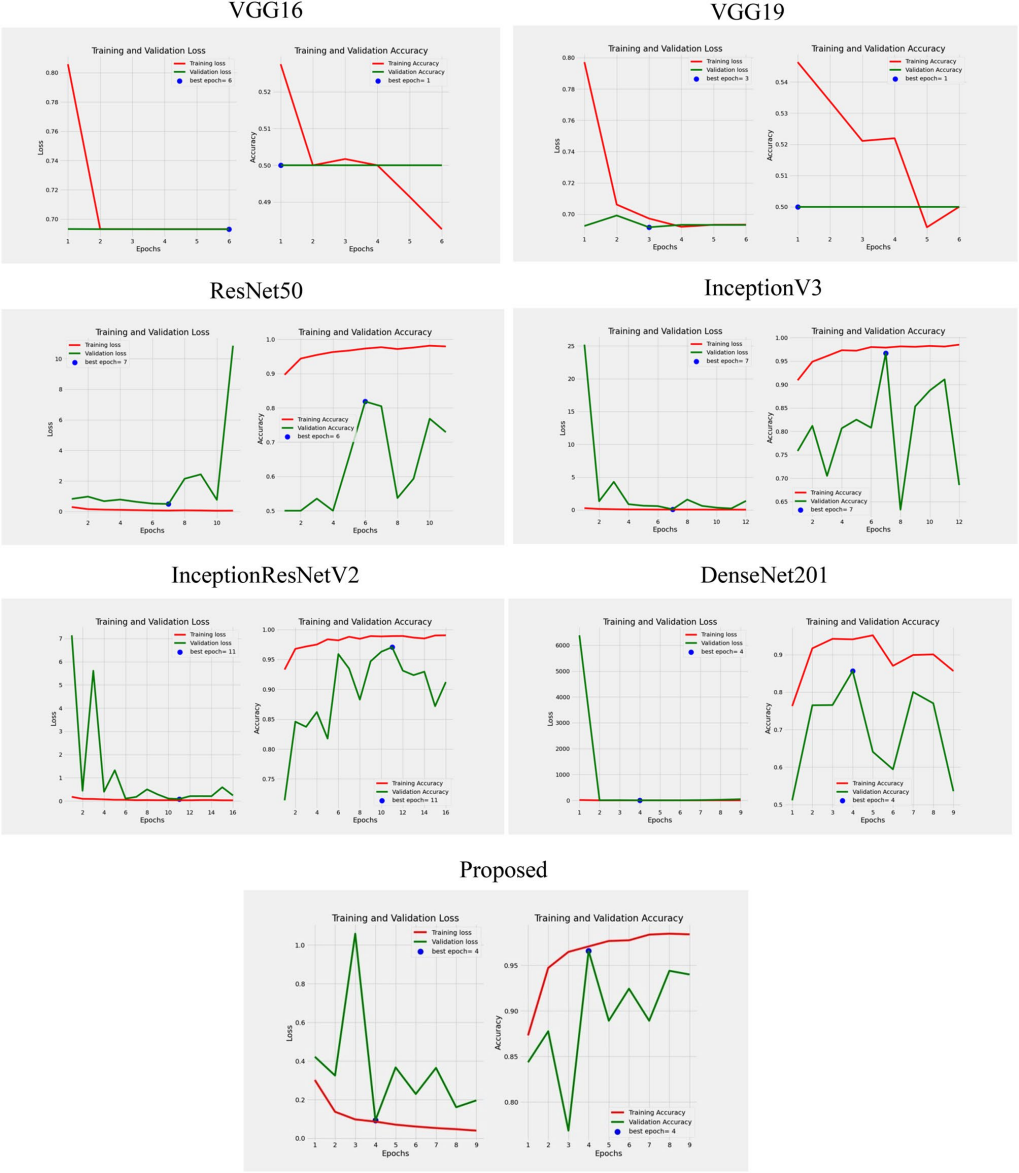
Comparative assessment of various deep learning models for classification of loom type.

**Figure 12:**
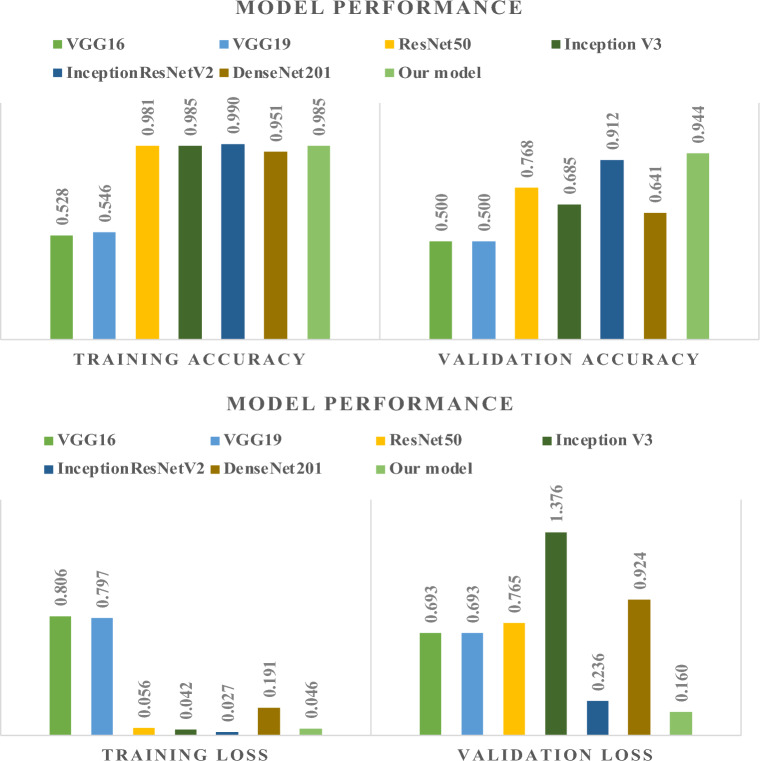
Accuracy and loss graphs.

To comment on the deep learning models’ generalization ability and to determine how well they are trained on the provided “*gamucha*” images, we first observed the accuracies as well loss values encountered with the training and validation test sets. The depiction is shown in Fig. 11. Considering each model’s accuracy represented by the graph, the ResNet50, InceptionV3, InceptionResNetV2, and DenseNet201 architecture demonstrate consistent and reliable training results in our experiments, although our proposed model is the best. However, validation accuracy is not comparatively good for other models except InceptionV3. Our model gives the validation accuracy. The equivalent curves are presented in Fig. 12 for clear demonstration.

As a potential next step to validate the results obtained so far, we utilized the other evaluation metrics mentioned in Section "Comprehensive overview of model characteristics". The related results are shown in Table 3. In terms of precision and recall, it is clearly seen that our model outperforms all other models. It signifies that our model predicts positive results with more correctness than the rest. It is important as the scenario of false negative in this case, i.e. predicting a powerloom “*gamucha*” as handloom has significant effect. Similarly, a high recall ensures that the model does not miss important instances of the positive class. Our model displays equally good recall as some which reveal high recall too.

**Table 3:** Performance metrics.

Model	Precision	F1-score	Sensitivity	Specificity
VGG16	0.000	0.000	1.000	0.000
VGG19	0.000	0.000	1.000	0.000
ResNet50	0.705	0.744	0.671	0.788
InceptionV3	0.614	0.761	0.371	1.000
InceptionResNetV2	0.965	0.764	0.977	0.633
DenseNet201	0.822	0.901	0.784	0.997
Our Model	0.895	0.943	0.883	0.997

After evaluating several pretrained Convolutional Neural Network (CNN) models, including DenseNet201, InceptionResNetV2, InceptionV3, and ResNet50, we noticed that while these models achieved respectable training accuracies, their performance on the validation dataset was notably lower than that of our proposed model. These models exhibited relatively lower validation accuracies and higher validation losses, indicating challenges in generalizing to unseen data for our specific task.

Furthermore, VGG16 and VGG19, which are well-established architectures, performed poorly on our classification problem, with validation accuracies close to random guessing (0.5). Additionally, these models tend to be computationally expensive, which raised concerns about practical deployment.

As a result, we decided to discard these pretrained models due to their limited ability to generalize effectively to our task, suboptimal performance, and computational inefficiency. Our proposed model emerged as the most suitable choice, offering superior performance, computational efficiency, and adaptability to our specific classification problem.

### Mobile application

**Figure 13:**
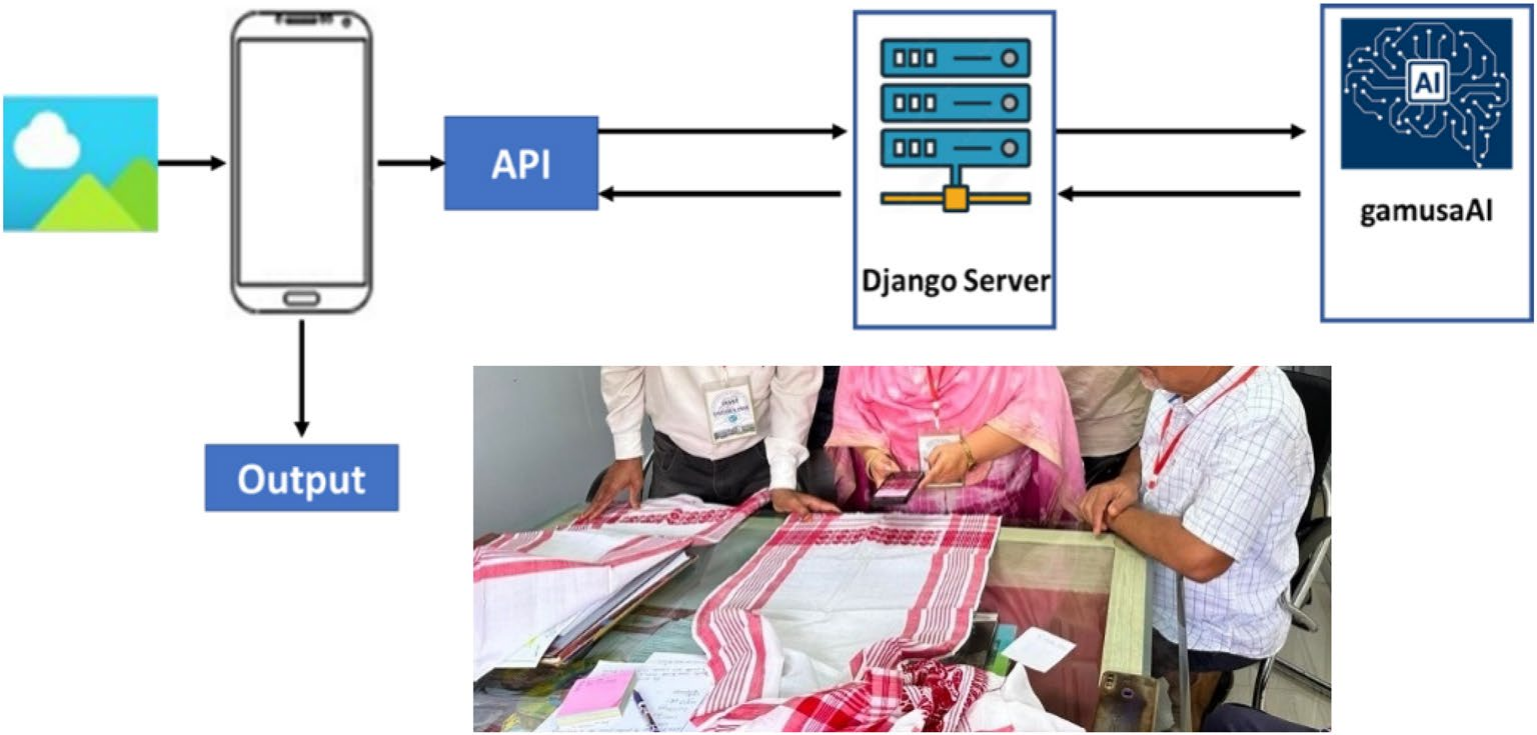
Workflow of the complete system; Testing of App in process.

**Figure 14:**
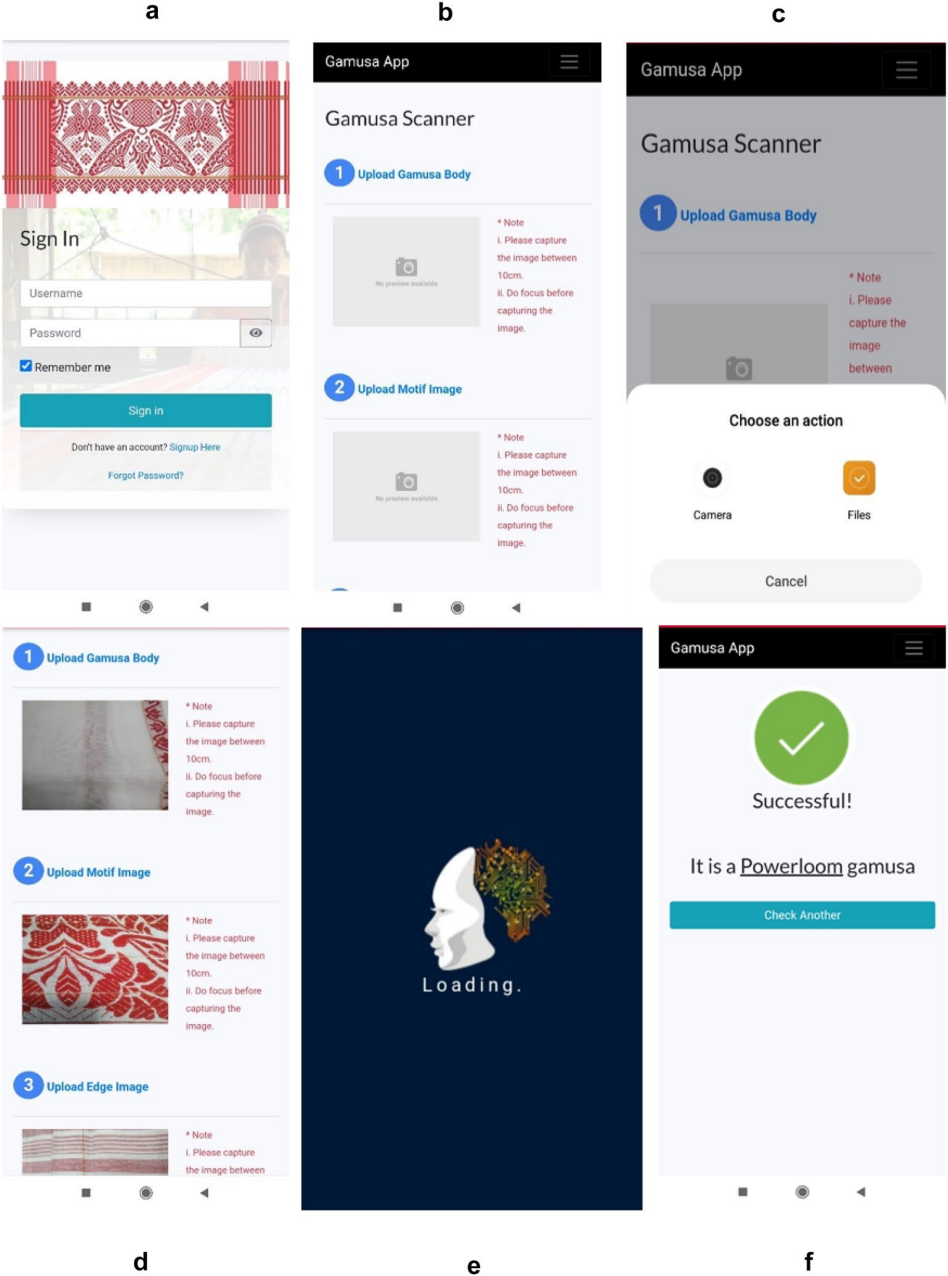
(**a**) Authentication, (**b**) The Main screen for uploading an image, (**c**) Capturing image (**d**) Uploading the images, (**e**) Processing, (**f**) Predicts the class after processing.

The complete workflow of the system with expert validation tests is shown in Fig. 13. Some screenshots of the Mobile Application Prototype and its flow are shown below Fig. 14.

## Conclusion

In conclusion, this study addresses the urgent need to preserve handloom traditions, focusing on the iconic “*gamucha*” towel from Assam, India. Despite its cultural and economic significance, the handloom industry faces challenges, including competition from powerloom counterparts. Deceptive practices exacerbate this crisis, impacting the livelihoods of weavers, especially female artisans. The study proposes an innovative AI-based solution to accurately identify handloom “*gamucha*”s amidst powerloom counterparts, aiming to combat misrepresentation in the market. By meticulously curating a benchmarked image dataset and developing a custom deep learning model, the study pioneers an automated recognition system capable of differentiating between handloom and powerloom “*gamucha*”s with high accuracy. The proposed model not only streamlines the identification process but also offers a user-friendly mobile application for seamless deployment, facilitating wider adoption and practical implementation.

Moreover, the study’s contributions extend beyond technological advancements, addressing broader societal and economic aspects. By safeguarding the integrity of handloom products and supporting the livelihoods of weavers, the research aligns with the goals of preserving cultural heritage and promoting sustainable practices in the textile industry. Additionally, the integration of AI technologies fosters transparency and trust within the supply chain, mitigating fraudulent practices and ensuring consumer confidence.

In essence, the study represents a holistic approach towards addressing contemporary challenges faced by the handloom industry, encompassing technological innovation, socio-economic empowerment, and cultural preservation. By embracing AI-driven solutions and fostering collaboration between traditional craftsmanship and modern technology, the research paves the way for a sustainable future for handloom traditions in the digital age.

## Data Availability

The datasets used and/or analysed during the current study available from the corresponding author on reasonable request.
